# A Meta‐Analysis and Systematic Review of the Effects of Sensory Modulation Treatments for Neurogenic Oropharyngeal Dysphagia

**DOI:** 10.1111/cns.70452

**Published:** 2025-07-08

**Authors:** Meng Dai, Ivy Cheng, Ayodele Sasegbon, Wanqi Li, Shaheen Hamdy

**Affiliations:** ^1^ Division of Diabetes, Endocrinology and Gastroenterology, School of Medical Sciences, Centre for Gastrointestinal Sciences, Faculty of Biology, Medicine and Health, Salford Royal Foundation Trust University of Manchester Manchester UK; ^2^ Rehabilitation Department The Third Affiliated Hospital of Sun Yat‐Sen University Guangzhou China; ^3^ Academic Unit of Human Communication, Learning, and Development, Faculty of Education The University of Hong Kong Hong Kong China; ^4^ Department of Rehabilitation Medicine, Guangzhou First People's Hospital Guangzhou Medical University Guangzhou China

**Keywords:** dysphagia, meta‐analysis, neurogenic, systematic review, treatment

## Abstract

**Background:**

Oropharyngeal sensory stimulation has been applied broadly in clinical dysphagia management, but evidence remains limited.

**Aims:**

We aimed to determine its effectiveness in treating neurogenic dysphagia (ND).

**Material and Methods:**

A systematic review and meta‐analysis was conducted using studies from PubMed, EMBASE (via Ovid), CINAHL, Web of Science, and the Cochrane Library, searched up to January 2025. We included randomized controlled trials (RCT) comparing sensory stimulations, including electrical and gustatory stimulation, with sham controls or placebo. The outcome measurements included swallowing scales based on clinical and instrumental evaluations.

**Results:**

We included 16 RCTs (620 participants) in the meta‐analysis. Overall, sensory stimulation significantly improved ND (*n* = 17, SMD [95% CI] = 0.80 [0.41, 1.20], *p* < 0.001; *I* ^2^ = 71%). Subgroup analysis revealed that the pooled effect size remained significant for electrical stimulation (*n* = 14, SMD [95% CI] = 0.79 [0.36, 1.23], *p* < 0.01; *I* ^2^ = 64%), but not for gustatory stimulation (*n* = 3, SMD [95% CI] = 0.76 [−1.68, 3.20], *p* = 0.31; *I* ^2^ = 90%). The pooled effect sizes for sensory stimulation were significant for both swallowing measurements (*n* = 14, SMD [95% CI] = 0.75 [0.27, 1.23], *p* < 0.01; *I* ^2^ = 76%) and acceleration of decannulation (*n* = 3, OR [95% CI] = 6.47 [1.10, 38.04], *p* = 0.05; *I* ^2^ = 3%).

**Conclusion:**

Oropharyngeal sensory stimulation improves swallowing function and decannulation in ND, with minimal adverse effects. While electrical stimulation shows clear benefits, gustatory effects remain inconclusive. Further studies are warranted to optimize protocols and confirm efficacy.

## Graphical abstract

This meta‐analysis systematically evaluated the effectiveness of oropharyngeal sensory stimulation in managing ND, demonstrating significant improvements in swallowing function and decannulation rates. While electrical stimulation exhibited consistent therapeutic benefits, the clinical implications of gustatory stimulation remain inconclusive and warrant further investigation.

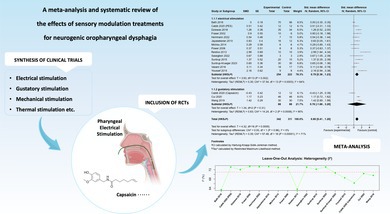

**Abbreviations**
ALS: amyotrophic lateral sclerosis
CN V: cranial nerve V (trigeminal nerve)
CN VII: cranial nerve VII (facial nerve)
CN IX: cranial nerve IX (glossopharyngeal nerve)
CN X: cranial nerve X (vagus nerve)
DSRS: Dysphagia Severity Rating Scale
EEG: electroencephalography
FEES: fibreoptic endoscopic evaluation of swallowing
fMRI: functional magnetic resonance imaging
fNIRS: functional near‐infrared spectroscopy
FOIS: Functional Oral Intake Scale
MS: multiple sclerosis
ND: neurogenic dysphagia
NTS: nucleus tractus solitarius
OD: oropharyngeal dysphagia
PAS: Penetration‐Aspiration Scale
PES: pharyngeal electrical stimulation
PSD: post‐stroke dysphagia
SSA: Swallowing Safety Assessment
TRP: transient receptor potential
VFSS: videofluoroscopic swallowing study

## 1 Introduction

Oropharyngeal swallowing is a complex physiological function that requires the integration of sensory and motor processes to facilitate the safe and efficient transfer of a bolus or saliva from the oral cavity and pharynx into the esophagus [1]. Swallowing is regulated by an intricate central network at multiple levels, encompassing cortical, subcortical, brainstem, and cerebellar structures [2]. Dysphagia frequently arises as a complication following various neurological conditions, such as stroke, traumatic brain injuries, brain tumors, and Parkinson's disease, among others. Neurogenic dysphagia (ND) refers to swallowing dysfunction resulting from neurological disease, and it can lead to dehydration, malnutrition, pneumonia, and even death, which will sharply impair the quality of life of those patients [3]. Generally, treatment for dysphagia can be categorized into functional recovery and compensation strategies targeting the peripheral and central nervous systems, with techniques like neurostimulation, peripheral stimulation, behavior training with or without biofeedback, and dietary modification [4].

Interventions targeting the peripheral or central sensorimotor nervous systems regulating swallowing could artificially harness the brain's natural propensity for plasticity after ND and affect overall swallowing function. Among those treatments, oropharyngeal sensation acts as an ideal target for impacting the whole swallowing system as the promoter. The sensory inputs travel via sensory fibers of cranial nerve V (CN V, trigeminal nerve), cranial nerve VII (CN VII, facial nerve), cranial nerve IX (CN IX, glossopharyngeal nerve), cranial nerve X (CN X, vagus nerve), synapsing at the brainstem level with cranial nerve nuclei and nucleus tractus solitarius (NTS). These inputs are further processed at the cortical level, including the insula and primary sensory cortex, thereby triggering and modifying swallowing motor responses in a feedforward and/or feedback manner [5]. Under physiological conditions, various forms of sensory inputs, including taste, viscosity, and volume of the bolus, could influence the biomechanics and safety of swallowing [6, 7, 8]. Partial or complete loss of oropharyngeal mucosal sensation is a crucial factor in developing ND after stroke and other neurological conditions [9, 10], which could lead to impaired secretion management and a delayed or absent swallowing reflex.

Clinical trials in this field have explored a variety of sensory stimulation interventions, including chemical, mechanical, thermal, and electrical stimulation of the oropharyngeal region, highlighting the increasing interest in sensory stimulation to enhance swallowing function for ND. However, the cumulative evidence for implementation in this field remains insufficient. The present systematic review and meta‐analysis synthesized the available evidence from published clinical trials, focusing on the effects of different sensory modulation treatments directly applied to the oropharyngeal region on outcomes in neurogenic oropharyngeal dysphagia.

## 2 Materials and Methods

This review adhered to the PRISMA (Preferred Reporting Items for Systematic Reviews and Meta‐Analyses) guidelines [11]. Two reviewers independently conducted the literature search and assessed the risk of bias. The first author extracted and synthesized the data, with the second author verifying the results. Any disagreements were resolved through consensus among all authors. The present meta‐analysis was registered in the INPLASY International Platform for Registered Systematic Reviews and Meta Analyses Program (Registration number: INPLASY202530031).

### 2.1 Study Identification and Search Strategy

**FIGURE 1:**
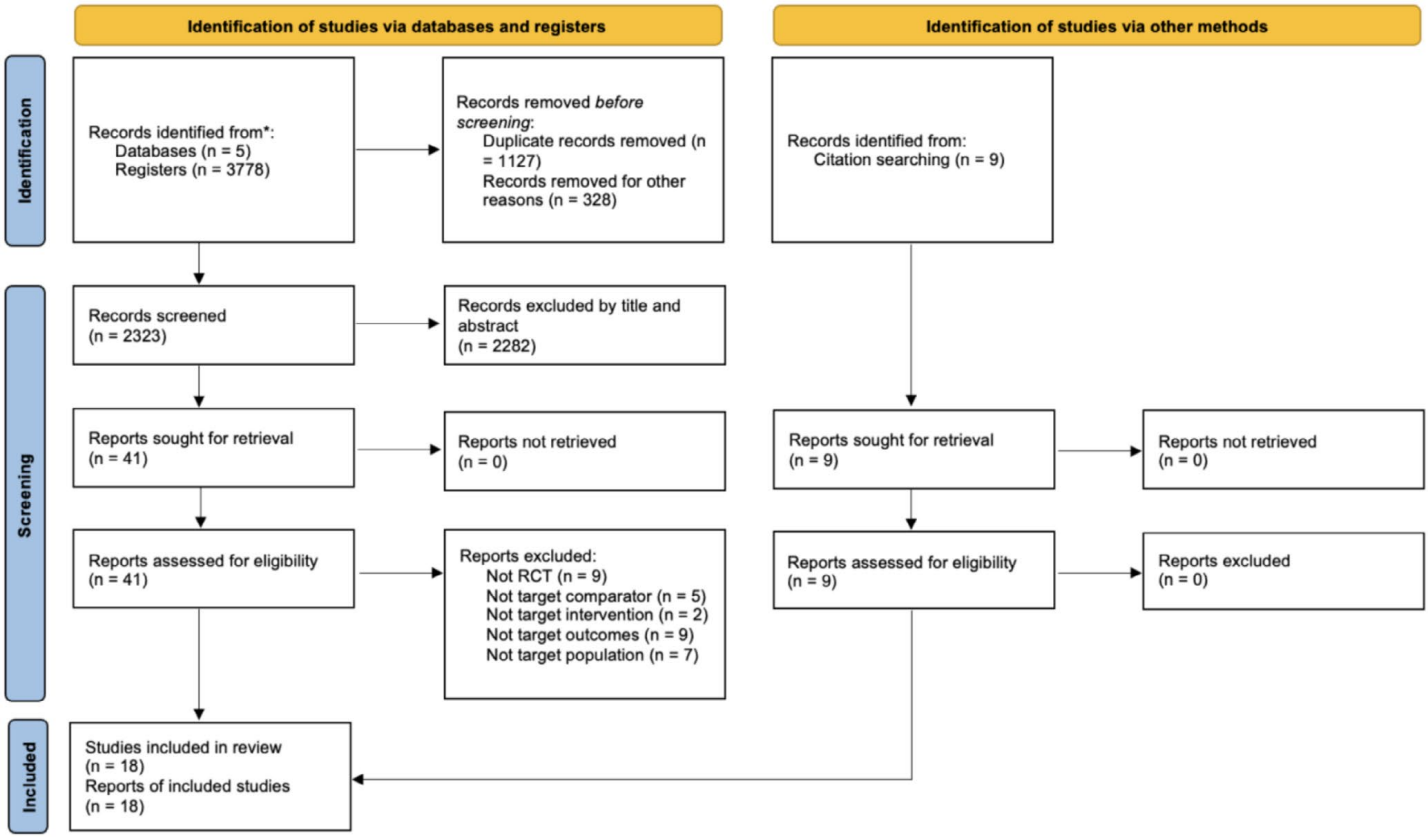
PRISMA 2020 flow diagram for systematic reviews including searches of databases, registers, and other sources.

A systematic search was conducted across five electronic databases from January 1970 to January 2025: PubMed, EMBASE (via Ovid), CINAHL, Web of Science, and the Cochrane Library. Reference checking and additional citation searching were also conducted, and relevant references from prior review articles were identified and noted as “identification of studies via other methods” in Figure 1. The key search terms were complied with the PICO strategy [12] with participants with ND were included, the intervention being oropharyngeal sensory stimulation, with control group needed to be present, with swallowing assessments performed. Detailed search strategies for each database can be found in Data S1.

### 2.2 Inclusion and Exclusion Criteria

Studies were eligible for analysis if they were randomized controlled trials (RCTs) including crossover, cluster‐RCTs, or quasi‐RCTs comparing oropharyngeal sensory stimulation as dysphagia treatment with either sham, no treatment, or minimal (standard care) intervention. Exclusion criteria included animal studies, case studies, open‐label trials, observational studies, retrospective analyses, studies lacking original data, and non‐English language publications.

### 2.3 Participants

Targeted participants were patients with neurogenic oropharyngeal dysphagia ‐ resulting from central or peripheral nervous system damage ‐ diagnosed clinically or through validated self‐report questionnaires, regardless of the timing of symptom onset. Studies with only healthy participants, patients without dysphagia, or those with dysphagia without neurogenic conditions, and dysphagia in elderly patients without any neurogenic diseases were excluded. For studies involving both dysphagic and non‐dysphagic patients and healthy participants, only data from the dysphagic group were extracted and analyzed.

### 2.4 Treatments

We included studies comparing oropharyngeal sensory stimulation with sham, placebo, or standard dysphagia therapy. Trials that combined multiple treatments (e.g., incorporating oropharyngeal sensory stimulation with other swallowing interventions) were eligible if study groups differed solely in applying the specific oropharyngeal sensory stimulation.

### 2.5 Outcomes

We included outcome measures related to swallowing, primarily functional evaluation scales based on clinical or instrumental assessments, as well as validated self‐reported dysphagia questionnaires. Studies that used decannulation and reintubation rates as a primary outcome measures were also included, as decannulation decisions were made based on the severity of dysphagia as a surrogate. However, studies applying non‐validated subjective ratings to assess swallowing ability were excluded. Additionally, data from the videofluoroscopic swallowing study (VFSS) quantitative measurements (i.e., timings only) were excluded due to variability in terminology definitions and uncertainty around thresholds for clinical benefit.

### 2.6 Data Extraction

The extracted data included the trial information (study design and trial registration), participant demographics (patient characteristics, age, and sample size), treatment details (treatment type, intensity, and duration), and outcome measures (reported as mean [standard deviation; SD] or mean [95% confidence interval; 95% CI]) and follow‐up protocols. For studies with multiple outcomes, the most clinically relevant swallowing‐related outcome was selected. For each outcome, we extracted the within‐group pre‐post mean difference (MD) and SD from baseline to the follow‐up results from the main outcome endpoint. If these values were unavailable, we extracted baseline and follow‐up measures to calculate the pre‐post MD and SD. When mean and/or SDs were missing, they were estimated using medians, quartiles, standard errors (SEs), confidence intervals (CIs), or *p*‐values. Transformation was conducted with online calculators using the quantile estimation (QE) method [13] (https://smcgrath.shinyapps.io/estmeansd/). For studies presenting data only in graphical format, two independent reviewers extracted data using WebPlotDigitizer 4.3 (https://apps.automeris.io/wpd/). The reliability and validity of WebPlotDigitizer for extracting graphed data have been previously established [14]. Before extraction, the tool was calibrated using the axis scales provided in each figure. The agreement between extractors was assessed, with disagreements resolved by consensus. The final values from the primary reviewer were used in the meta‐analysis after confirming acceptable agreement. When data were unavailable, we contacted the corresponding authors. Studies were excluded if data could not be obtained through the above methods.

### 2.7 Risk of Bias Assessment

The Cochrane Risk of Bias 2 (ROB 2) tool was applied, and figures were generated using the ROB 2 assessment tool (ROB 2 IRPG Beta V9) [15]. The risk of bias in RCTs was assessed as low, some, or high concerns across five domains: D1—bias arising from the randomization process; D2—bias due to deviations from intended interventions; D3—bias due to missing outcome data; D4—bias in the measurement of the outcome; and D5—bias in the selection of the reported result following the Cochrane handbook [16].

### 2.8 Statistical Analysis/Data Synthesis

Statistical analyses were conducted using Review Manager online (https://revman.cochrane.org/info). Treatment effect sizes were calculated by comparing treatment outcomes to those of the control groups. Data used for calculating treatment effects included group sizes, MDs (Mean_pre‐treatment_ − Mean_post‐treatment_), and pooled SDs. The pooled SD was calculated using a standard formula [17].

$$SD_{\text{pooled}}=\sqrt{\frac{\left(n_{\mathrm{pre}}-1\right)SD_{\mathrm{pre}}^{2}+\left(n_{\text{post}}-1\right)SD_{\text{post}}^{2}\;}{n_{\mathrm{pre}}+n_{\text{post}}-2}}$$

For outcome measures that increased with disease severity, mean values were multiplied by ‐1 to standardize directionality [15]. For the combination of the dichotomous data and continuous data, the following formulas were applied to switch odds ratio (OR) to standardized mean differences (SMD) and SE(OR) to SE(SMD), SE was calculated based on the upper/lower limit of 95% CI [18].

$$\mathrm{SMD}=\text{In}\left(\text{OR}\right)∙\frac{\sqrt{3}}{\pi }$$

$$\mathrm{SE}\left(\text{OR}\right)=\frac{\text{In}\left({\mathrm{CI}}_{\text{high}}\right)-\text{In}\left({\mathrm{CI}}_{\mathrm{low}}\right)}{2∙Z}\;\left(Z=1.96\right)$$

$$\mathrm{SE}\left(\mathrm{SMD}\right)=\mathrm{SE}\left(\text{OR}\right)∙\frac{\sqrt{3}}{\pi }$$

Combined outcomes were analyzed based on subgroups of electrical stimulation and gustatory stimulation using SMD with SE, and a random‐effects model based on inverse variance was applied to compute a weighted average SMD across studies. Statistical significance was set at *p* < 0.05, and effect sizes were reported as SMD (95% CI). Effect size magnitude was interpreted following Cohen's criteria, where an SMD of 0.2 indicated a small effect, 0.5 a medium effect, and 0.8 a large effect. Heterogeneity was evaluated using Cochrane's *Q* statistic and the *I* ^2^ test [19], with low heterogeneity defined as *I* ^2^ between 0% and 25%, moderate heterogeneity defined as *I* ^2^ between 25% and 50%, substantial heterogeneity defined as *I* ^2^ between 50% and 75%, and considerable heterogeneity defined as *I* ^2^ > 75%. A leave‐one‐out analysis was employed to understand the impact of each study on the overall pooled effect estimate and the heterogeneity using the metafor package [20] from R (StataCorp, College Station, TX, USA). Sensitivity analysis was conducted using a sequential approach based on leave‐one‐out analysis results. We systematically removed a minimum number of studies that were identified as the primary contributors to statistical heterogeneity stepwise until a moderate heterogeneity (*I* ^*2*^ < 50%) was reached with remaining studies.

## 3 Results

### 3.1 Study Characteristics

A total of 3778 records were retrieved from five databases. After removing 1127 duplicates, 328 records for other reasons, 2323 records remained for title and abstract screening. Additionally, nine articles were identified through other methods. After screening, 50 articles were selected for full‐text review to assess eligibility. Among these, 32 articles were excluded due to the following reasons: non‐RCT design (*n* = 9), inappropriate comparator (*n* = 5), non‐target intervention (*n* = 2), non‐target outcomes (*n* = 9), or non‐target population (*n* = 7). In total, 18 articles were included in the systematic review (Figure 1).

Of the 18 published studies included in this review, 12 focused on post‐stroke patients (*n* = 587 participants [pts]), one on patients with Parkinson's disease (*n* = 3 pts), three on tracheostomized patients with severe post‐stroke dysphagia (PSD) (*n* = 159 pts), one on dysphagia patients with amyotrophic lateral sclerosis (ALS) (*n* = 20 pts), and one on dysphagia patients with multiple sclerosis (MS) (*n* = 20 pts). Most studies used a parallel RCT design (15 trials, 78%), and 3 used a crossover RCT design where patients served as their own control (detailed information illustrated in Table 1).

**TABLE 1:** Study characteristics of included studies.

Source	Trial information		Baseline participant characteristics			Interventions			Outcome measures		
Author, year	Study design	Registration no.	Patient characteristics	Sample size (n, females)	Age mean (SD), years	Treatment	Duration	Comparison	Swallowing‐related outcome	Follow‐up schedule	Included/Not in the meta‐analysis
**Electrical**											
Bath et al. 2016 [21]	RCT, parallel	ISRCTN25681641	Patients with a recent ischemic or hemorrhagic stroke and dysphagia	162 (68)	74.4 (11.2)	PES	10 min per day 3 days	Sham PES	PAS, PAS > 3, QOL, AE	Baseline, 2 weeks, 6 weeks, and 12 weeks	Y
Cabib et al. 2020 [22]	RCT, cross‐over	NCT04052178	Patients with unilateral stroke and chronic unsafe swallow	12 (1)	70.0 ± 14.2	PES	10 min	Potassium sorbate/Sham stimulation	PAS and VFSS timing measurements: LVC UESO	Immediate effects	Y
Dziewas et al. 2018 [23]	RCT, parallel	ISRCTN18137204	Tracheotomy patients with a supratentorial stroke and could not be decannulated because of severe dysphagia	69 (25)	pes group *n* = 35 61.7 (13.0) Sham group *n* = 34 66.8 (10.3)	PES	10 min per day 3 days	Sham PES	DSRS FOIS	Baseline, day 2, day 10, day 30 and day 90	Y
Essa et al. 2017 [24]	RCT, parallel	ISRCTN83103698	Dysphagic stroke patients	36 (13)	Active group 58.6 (13.42) Sham group 70.5 (11.8)	PES	10 min per day 3 days	Sham PES	DSRS	Baseline, 2 weeks and 3 months	N
Everton et al. 2021 [25]	RCT, parallel	ISRCTN25681641	Patients with a recent ischemic or hemorrhagic stroke and dysphagia	81 (34)	73.4 (11.2)	PES	10 min per day 3 days	Sham PES	Eight VFSS timing measurements, including STD and PTT, clearance measures comprised oral and pharyngeal residue and swallows to clear	Baseline and 2 weeks	N
Fraser et al. 2002 [26]	RCT, parallel	No registration information	Sixteen dysphagic, acute hemispheric stroke patients	16 (6)	74.2 (9.0)	PES	5 Hz for 10 min	Sham PES	PAS and VFSS timing measurements: SRT, PTT	Before and 60 min after the intervention	Y
Herrmann et al. 2022 [27]	RCT, parallel	NCT03481348	ALS patients with severe dysphagia (characterized by a PAS of at least 4 in thin liquid)	20 (12)	PES group: 76.0 (8.9) Control group: 57.5 (13.3)	PES	10 min per day 3 days	Sham PES	PAS, SWAL‐QOL, DSRS, residue	Baseline, 1 day, 4 days, 3 weeks and 3 months after treatment	Y
Jayasekeran et al. 2010 [28]	RCT, parallel	ISRCTN83103698	Dysphagia patients after anterior circulation cerebral infarct or hemorrhage	28 (9)	75 (2)	PES	10 min per day 3 days	Sham PES	DSRS, PAS, feeding status, hospitalization	Baseline 2 weeks after first intervention	Y
Michou et al. 2014 [29]	RCT Sham controlled cross‐over	ISRCTN83103698	Stroke patients with dysphagia which persisted for more than 6 weeks postictus	6 (1)	51,66	PES	5 Hz for 10 min	Sham PES	Cumulative PAS, and VFSS timing measurements: OTT, PRT, PTT, AC, UOD	Before and after the intervention	Y
Power et al. 2006 [30]	RCT, parallel	No registration information	Hemispheric stroke patients, with a diagnosis of dysphagia	16 (4)	73 (12)	Faucial pillar electrical stimulation	10 min	Sham stimulation	Cumulative PAS score, and VFSS timing measurements: OTT PTT SRT LCD UOD	Before and 60 min after the treatment	Y
Restivo et al. 2013 [31]	RCT, parallel	No registration info	MS patients with swallowing difficulty for liquids, solids or both, present for at least two consecutive months	20 (13)	39.7 (6.5)	PES	10 min per day 5 days * 4 weeks	Sham PES	PAS, EMG measurement	Before (T0) and immediately (T1), 2 weeks (T2), and four (T3) weeks after the last session	Y
Sasegbon et al. 2022 [32]	RCT, cross‐over	NCT03253354	Patients with PD	3 (0)	75 (±12)	PES	5 Hz for 10 min	Sham PES	Cumulative PAS score, and VFSS timing measurements: OTT, PTT, PRT from VFSS recording	Baseline and post‐interventional VFSS recordings	Y
Suntrup et al. 2015 [33]	RCT, parallel	NCT01956175	Post‐stroke patients who were tracheotomized and suffered from severe persistent dysphagia	30 (15)	PES group 63.0 (14.5) Control group 66.7 (14.5)	PES	10 min per day 3 days	Sham PES	Whether they could be decannulated or not, feeding status assessed at discharge with FOIS, mRS, LOS on ICU/in the hospital, and time from stimulation to discharge	Baseline, 3 days, and at discharge	Y
Suntrup‐Krueger et al. 2023 [34]	RCT, parallel	NCT02470078	Extubated acute stroke patients with severe dysphagia, defined as a score of > 4 on the validated 6‐point fibreoptic endoscopic dysphagia severity scale (FEDSS)	60	Not mentioned	PES	10 min per day 3 days	Sham PES	Reintubation feeding status	Baseline and 120 h	Y
Vasant et al. 2016 [35]	RCT, parallel	ISRCTN83103698	Stroke Patients with new‐onset dysphagia following anterior or posterior cerebral circulation, within 6 weeks	36 (14)	71 (60, 79)	PES	10 min per day 3 days	Sham PES	DSRS, unsafe swallowing based on PAS, removal of NGT, hospital discharge	Baseline, 2 weeks and 3 months	Y
Youssef et al. 2015 [36]	RCT, parallel	No registration information	Acute severely dysphagic stroke patients	18 (5)	PES group 64.3 ± 11.3 Sham group 66.7 ± 8.5	PES	10 min per day 3 days	Sham stimulation	PAS FOIS, pharyngeal secretion, pharyngeal stasis, patient global satisfaction	Baseline and 2 weeks	Y
**Gustatory**											
Cabib et al. 2020 [22]	RCT, sham‐controlled cross‐over	NCT04052178	Patients with unilateral stroke and chronic unsafe swallow	12 (5)	74.3 ± 7.8	oral capsaicin	Capsaicin 10^−5^ M were administered once in a 100 mL solution	Potassium sorbate/Sham stimulation	PAS, and VFSS timing measurements: LVC, UESO	Immediate effects	Y
Cui et al. 2020 [37]	RCT, parallel	No registration information	Patients with dysphagia after stroke	92 (35)	59.11 ± 7.84 (Experiment group) 58.35 ± 7.01 (Control group)	combined capsaicin and ice stimulation	Twice a day, 20 min before lunch and dinner, for a total of 3 weeks	An ice (containing normal saline solution) swab	Grade of WST; SSA scores	Baseline and 3 weeks	Y
Wang et al. 2019 [38]	RCT, parallel	[2018]‐(K015)	Hospitalized stroke patient	60 (24)	63.77 (12.18) (treatment) 66.23 (11.91) (control)	thermal tactile stimulation with natural capsaicin	3 weeks	The solution and the nectar bolus were made to be identical to that in the intervention group, except for capsaicin	VVST, EAT‐10, SSA	Baseline and 3 weeks	Y

*Note:* Cabib et al. explored both the capsaicin and PES in one trial; thereby, the data were both included for electrical stimulation and gustatory stimulation.

Abbreviations: AC, airway closure time; AE, adverse events; ALS, amyotrophic lateral sclerosis; CNS, central nervous system; CTL, carbonated thin liquids; CTL, carbonated thin liquids; DSRS, Dysphagia Severity Rating Scale; DST, duration of stage transition; EAT‐10, Eating Assessment Tool; EMG, electromyography; FOIS, Functional Oral Intake Scale; ICU, intensive care unit; LOS, length of stay; LVC, laryngeal vestibule closure time; mRS, modified Rankin Scale; MS, multiple sclerosis; NGT, nasogastric tube; OTT, oral transit time; PAS, penetration and aspiration scale; PES, pharyngeal electrical stimulation; PRT, pharyngeal response time; PTT, pharyngeal transit time; QOL, quality of life; RCT, randomized controlled trial; SRT, swallow response time; SSA, Standard Swallowing Assessment; STD, stage transition duration; SWAL‐QOL, swallowing quality of life questionnaire; TSD, total swallow duration; UOD/UESO, upper esophageal sphincter opening time; VFSS, videofluoroscopic swallowing study; V‐VST, volume‐viscosity swallow test; WST, water swallowing test.

### 3.2 Treatment

The oropharyngeal sensory stimulation in all included RCTs can be categorized as electrical stimulation in the pharynx (15 studies) and faucial pillar (1 study), and gustatory stimulation with capsaicin (3 studies). Cabib et al. [22] explored both the capsaicin and pharyngeal electrical stimulation (PES) in one clinical trial; therefore, the data were separated and extracted for two interventions. Detailed information is provided in Table 1.

In most PES studies, treatment was administered for 10 min daily over three consecutive days, which has been found to be the optimal protocol in a dose–response study, except in one study where a 5‐day protocol was implemented [31]. Three other PES studies explored the instant effect after one 10‐min PES session [26, 29, 32]. Power et al. [30] investigated oral stimulation at the faucial pillar with a single 10‐min session. For gustatory interventions, treatment patterns were more variable. Two studies applied capsaicin solution soaked ice swabs multiple times daily for 3 weeks [37, 38], while three studies respectively administered capsaicin solution [22] once and measured the immediate effects post‐intervention during the VFSS examination.

### 3.3 Outcome Measures and Follow‐Up Schemes

Most studies utilized both clinical evaluation and/or instrumental assessment, primarily VFSS and fibreoptic endoscopic evaluation of swallowing (FEES) to assess swallowing function. The outcome measures included the Penetration–Aspiration Scale (PAS) (11 studies), Dysphagia Severity Rating Scale (DSRS) (5 studies), the Swallowing Safety Assessment (SSA) (2 studies), and the Functional Oral Intake Scale (FOIS) (3 studies). Seven studies also incorporated various temporal measurements derived from VFSS that were not used for our analysis.

In terms of follow‐up duration, the primary outcomes were mostly the swallowing measurements around 2 weeks after the intervention, with the longest reaching up to 3 months. Additionally, some studies focused on observing the immediate effects of these interventions within an hour. For two studies on the effects of PES on decannulation, the primary endpoint of the study was readiness for decannulation after 3 days of PES treatment, assessed with the FEES‐based algorithm [39]. The most recent of these studies set the need for reintubation within 120 h of extubation as the primary endpoint.

### 3.4 Risk of Bias Assessment

**FIGURE 2:**
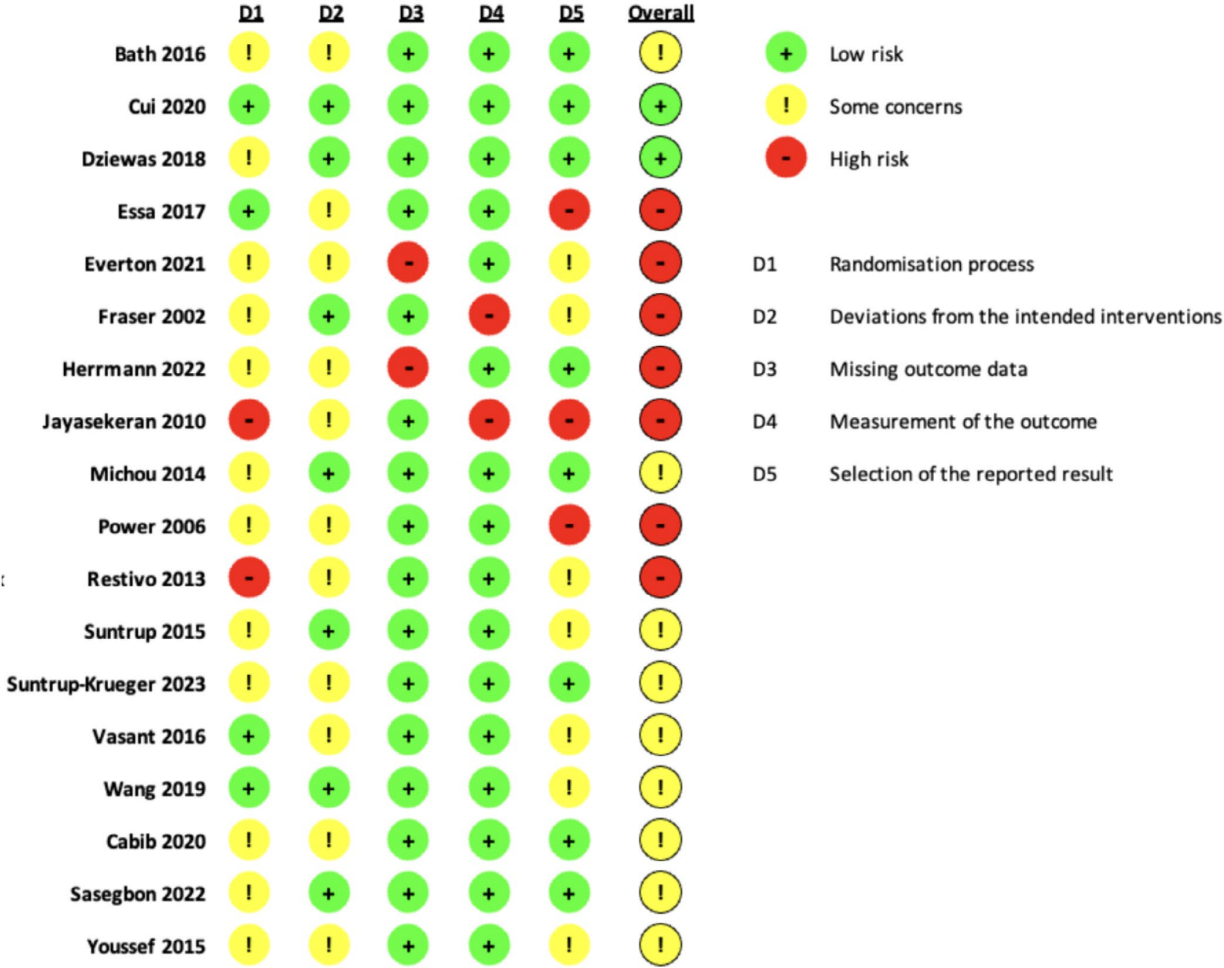
Risk of bias summary for individual studies.

**FIGURE 3:**
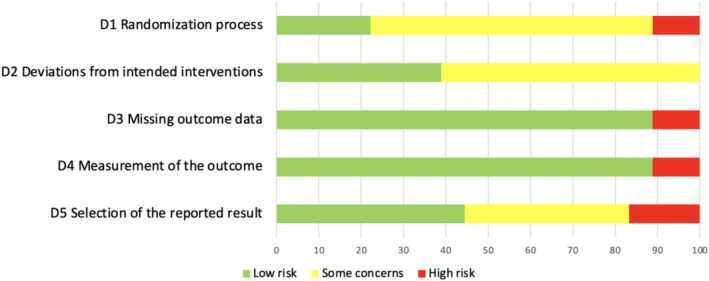
Risk of bias graph for all included studies.

The risk of bias assessment is summarized in Figures 2 and 3. Eighteen trials were assessed (7 high risk, 9 some concerns, and 2 low risk). The primary concerns included issues with the randomization process, deviations from intended interventions, and selection of the reported result. These concerns were related to the lack of reporting on the concealment of intervention, the difficulty with implementing double‐blindness procedures, and discrepancies between reported outcomes and original registration plans. Most studies were evaluated as low risk for measurement of the outcome and missing outcome data.

### 3.5 Meta‐Analysis of the Effects of Oropharyngeal Sensory Modulation on ND

Two studies were further excluded from the meta‐analysis due to the fact that they were secondary analyses of reduplicative clinical trials [24, 25]. Thus, in total, 16 RCTs with 620 participants were included in the meta‐analysis. One study applied both PES and capsaicin as treatment groups; therefore, it was analyzed under both categories, leading to the final analyzed dataset between the experimental group and control group, being 17 studied groups in total (Figure 1).

**FIGURE 4:**
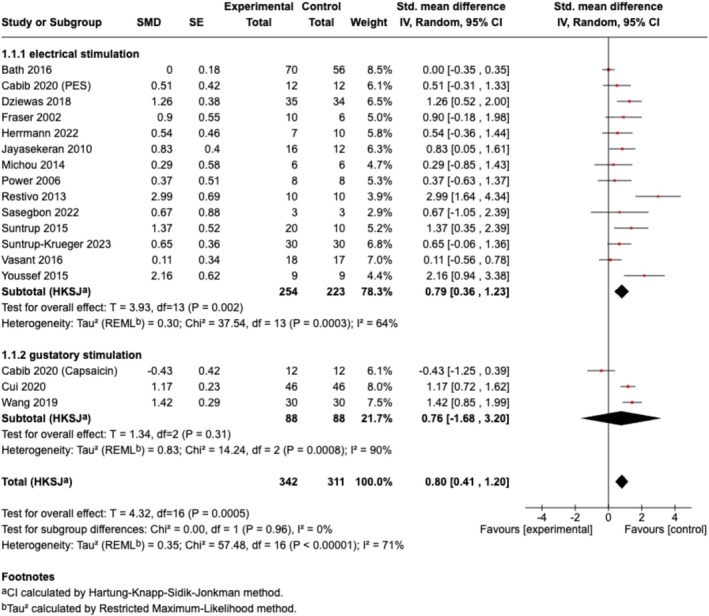
Forest plot of the combined meta‐analysis from both continuous data (swallowing measurements) and dichotomous data (decannulation/reintubation). Subgroup analysis was shown under subgroups electrical stimulation (1.1.1) and gustatory stimulation (1.1.2). (1) After screening for eligibility, only studies that applied capsaicin‐based interventions fulfilled all inclusion and exclusion criteria for this review. Therefore, in the subgroup “gustatory stimulation”, only capsaicin‐based interventions were analyzed. (2) Cabib et al. explored both the capsaicin and PES in one trial; thereby, the data were both included for electrical stimulation and gustatory stimulation.

**FIGURE 5:**
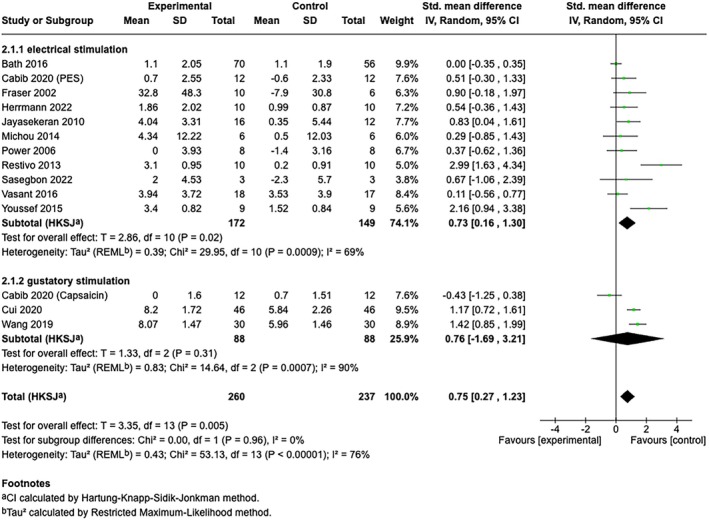
Forest‐plot of the meta‐analysis for oropharyngeal sensory stimulation on continuous data from swallowing outcome measures. Subgroup analysis was shown under subgroups electrical stimulation exercises (2.1.1) and sensory stimulation (2.1.2).

**FIGURE 6:**
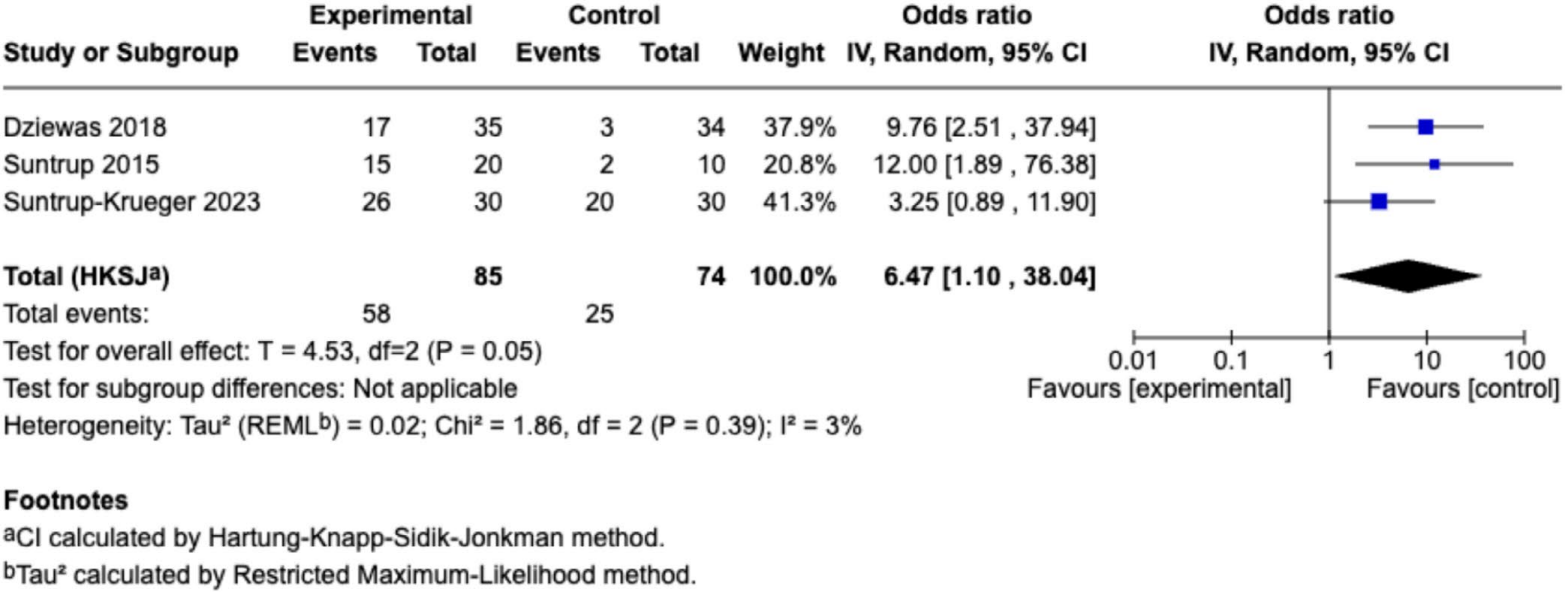
Forest‐plot of the meta‐analysis for oropharyngeal sensory stimulation on dichotomous data for readiness for decannulation or the extubation failure rate.

The overall pooled effect size of all included sensory stimulations on ND was large and significant (*n* = 17, SMD [95% CI] = 0.80 [0.41, 1.20], *p* < 0.001; *I* ^*2*^ = 71%). In the subgroup analysis for different sensory stimulation strategies, electrical stimulation showed a significant effect size with substantial heterogeneity (*n* = 14, SMD [95% CI] = 0.79 [0.36, 1.23], *p* < 0.01; *I* ^*2*^ = 64%), while gustatory stimulation with capsaicin yielded a non‐significant effect size with high heterogeneity (*n* = 3, SMD [95% CI] = 0.76 [−1.68, 3.20], *p* = 0.31; *I* ^*2*^ = 90%) (Figure 4). Further subgroup analyses based on outcome categories indicated that the pooled effect sizes of sensory stimulation were significant for continuous data for swallowing measurements (*n* = 14, SMD [95% CI] = 0.75 [0.27, 1.23], *p* < 0.01; *I* ^*2*^ = 76%) (Figure 5) and borderline significant for dichotomous data for readiness for decannulation (*n* = 3, OR [95% CI] = 6.47 [1.10, 38.04]; *p* = 0.05; *I* ^*2*^ = 3%) (Figure 6).

### 3.6 Sensitivity Analysis

**FIGURE 7:**
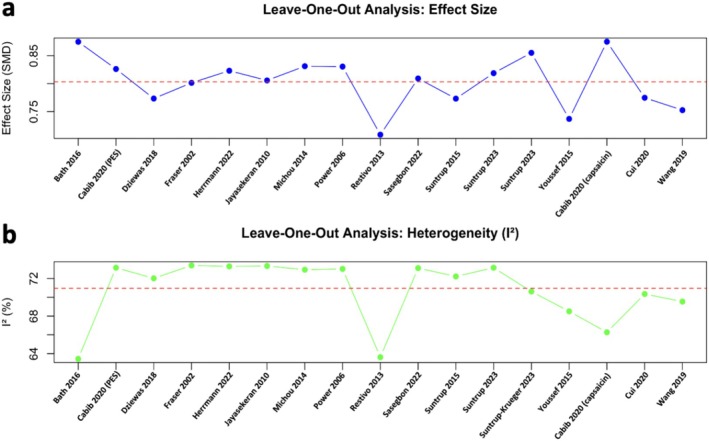
Leave‐one‐out analysis. (a) leave‐one‐out analysis for pooled effect size with the value at each point indicating the effect size by excluding the study, (b) leave‐one‐out analysis for heterogeneity with the value at each point indicating the *I* ^2^ by excluding the study.

The leave‐one‐out analysis for effect size showed that the pooled effect size (SMD) remains relatively stable, indicating that no single study disproportionately affects the overall results. However, Restivo et al. (2013) and Youssef et al. (2015) [31, 36] led to slightly more shifts in the effect size when excluded (Figure 7a). Bath et al. (2016), Restivo et al. (2013), and Cabib et al. (2020) [21, 22, 31] were identified as the primary contributors to statistical heterogeneity (Figure 7b).

**FIGURE 8:**
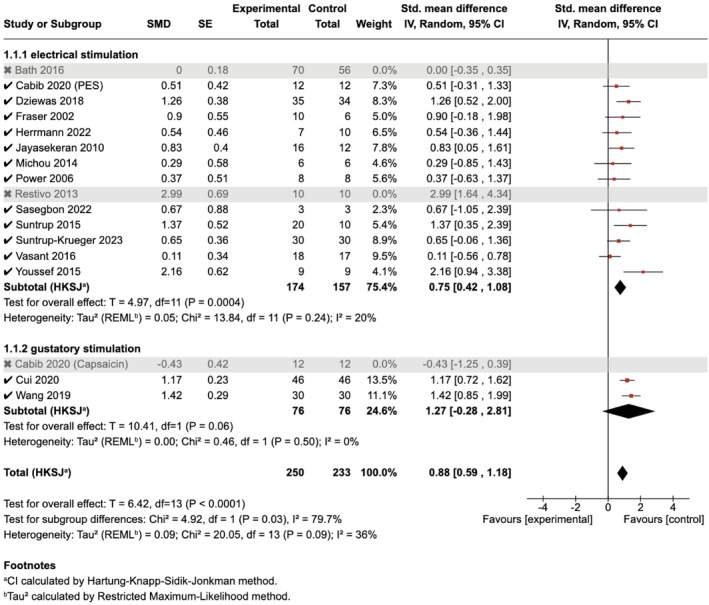
Sensitivity test after excluding 3 studies for the combined meta‐analysis.

Based on the results of the impact of each study on the heterogeneity, we further attempted to exclude the minimum number of studies to improve the heterogeneity of the meta‐analysis. Only by excluding three studies, namely Restivo et al. (2013), Bath et al. (2016), and Cabib et al. (2020) (capsaicin) [21, 22, 31], were we able to reach a low heterogeneity (*I* ^*2*^ = 36%); however, the overall pooled effect sizes of the sensory stimulations on ND remained significant (*n* = 14, SMD [95% CI] = 0.88 [0.59, 1.18], *p* < 0.001). The effect size of the subgroup analysis changed accordingly after excluding the three datasets. Both subgroups exhibited low heterogeneity: the electrical stimulation group showed significant effect sizes (*n* = 12, SMD [95% CI] = 0.75 [0.42, 1.08], *p* < 0.001; *I* ^*2*^ = 20%) and the gustatory stimulation group showed non‐significant effect sizes (*n* = 2, SMD [95% CI] = 1.27 [‐0.28, 2.81], *p* = 0.06; *I* ^*2*^ = 0%) (Figure 8).

### 3.7 Adverse Events

Overall, 10 articles reported or mentioned relevant adverse events. PES was generally well‐tolerated among most patients, with 3 studies reporting minor discomfort in the pharynx [22, 23, 27]. In a study focusing on post‐stroke tracheostomy patients, a high rate of adverse events was observed (69% in the PES group and 71% in the sham group), and deaths were reported, but none of the serious adverse events were attributed to PES itself.

## 4 Discussion

This systematic review and meta‐analysis summarizes the current applications of oropharyngeal sensory stimulation for treating ND. The results demonstrated significant overall effects on both swallowing function and decannulation among patients receiving oropharyngeal electrical stimulation and gustatory stimulation (capsaicin). However, rather high heterogeneity was observed among studies, and the sample sizes for included RCTs were rather small, especially for capsaicin studies. As such, the findings should be interpreted with some caution. Adverse events related to sensory interventions were rarely reported and mostly included minor discomfort in the pharynx. Additionally, the overall sample size of the relevant clinical trials remains small, which may affect the generalizability of our findings. This meta‐analysis provides cumulative evidence supporting the potential efficacy of sensory modulation interventions, paving the way for larger, high‐quality studies to validate their clinical application and explore long‐term outcomes.

### 4.1 Comparisons With Other Studies

Previous publications have explored oropharyngeal sensory stimulation for ND. Consistent with our findings, an individual patient data meta‐analysis summarized the results from three trials on PES and found it to be safe and effective, improving aspiration, overall dysphagia severity, and possibly reducing the length of hospital stay [40]. A systematic review of neuromodulation on PSD, which included PES, showed an overall moderate effect size (SMD [95% CI] = 0.68 [0.22, 1.14], *p* = 0.004) [41]. Similarly, a more recently published meta‐analysis on PES reported significant therapeutic effects on swallowing functions in PSD, although no significant positive effects on oral ingestion, reduction of aspiration, or length of hospitalization were observed [42]. By contrast, one meta‐analysis that explored intraoral treatments, including oromotor exercises and sensory stimulation, found no significant benefits for neurogenic oropharyngeal dysphagia [43]. Another meta‐analysis on the effect of capsaicin suggested that capsaicin and PES together had significant therapeutic effects for oropharyngeal dysphagia [44]. Also, capsaicin, as one of the transient receptor potential (TRP) channel agonists, showed a large pooled effect size compared to placebo interventions (SMD [95% CI] =1.27[0.74, 1.80], *p* < 0.001; *I* ^2^ = 79%) for neurogenic (and aging related) oropharyngeal dysphagia [45]. In our subgroup analysis, we did not observe significant results while including three studies with capsaicin. However, a significant effect size was noticed after excluding Cabib et al. (2020) in sensitivity analysis [22].

The observed discrepancies across results may be due to the following factors. First, the treatment and the intervention time differed between studies. In the meta‐analysis for intraoral treatments, there are four studies concentrating on motor exercises targeting the lips and tongue, and one study on electrical stimulation of the oral cavity, which differed from the current meta‐analysis. Furthermore, some studies, such as that by Cabib et al. (2020) [22], have implemented only a single treatment session, which may contribute to negative outcomes.

Second, the participants differed across the recruited studies. In the current meta‐analysis, we included patients with ND of various etiologies while excluding geriatric dysphagia. In contrast, previous meta‐analyses have either focused solely on PSD patients or included elderly dysphagia patients. Sensory deficits play a key role in ND, as they are linked to a higher prevalence of aspiration. Oropharyngeal sensory stimulation may act more effectively for ND by compensating for the loss of sensitivity [9]. Geriatric dysphagia, however, involves more complex age‐related changes, such as muscle loss, reduced tissue elasticity, diminished sensory function, decreased saliva production, and compromised brain capacity, compounded by frailty and functional impairments [46].

Third, the included studies differed. As compared with the previous meta‐analysis for TRP channel agonists, we excluded the studies solely focusing on OD associated with aging and the aural or nasal capsaicin stimulation studies. The differing results reveal the current shortage of evidence for capsaicin among OD with neurogenic diseases.

### 4.2 Heterogeneity Among Studies and Current Research Status

Considerable heterogeneity was observed among the studies. Excluding studies by Bath et al. (2016), Restivo et al. (2013), and Cabib et al. (2020) [21, 22, 31] reduced the heterogeneity to 36%, while the overall effectiveness remained significant. This suggests that the majority exhibit consistently low variability.

Several factors may account for this heterogeneity. First, differences in outcome measures were evident. Among the included RCTs, five used PAS as the primary outcome measure, four used DSRS, and two used SAS. Although these evaluations are well‐established with stable sensitivity and specificity for assessing swallowing functions, their differing focuses may contribute to the observed variability. PAS [47], which measures bolus airway intrusion, emphasizes immediate safety impairments observed during instrumental examinations like VFSS or FEES. In contrast, DSRS [48] and SAS [49] are clinically evaluated scores based on overall oral intake status. Additionally, the analysis of PAS is variable: some studies report the highest score for a single bolus, while others calculate an average from multiple boluses, potentially biasing the results when reflecting overall swallowing function.

Second, intervention doses varied across studies. For instance, while Cabib et al. and Power et al. [26, 32] investigated immediate treatment effects, most other studies implemented protocols involving 10‐min daily interventions for 3 days.

Third, there were differences in patient groups. While most studies focused on PSD, Herrmann et al. evaluated treatment effects in ALS patients [24], and Restivo et al. included patients with MS [27]. The severity of dysphagia and episode time for diseases also varied, with some studies focusing on tracheostomy patients experiencing severe dysphagia post‐stroke, while Bath et al. recruited patients in the acute phase, where neural plasticity may differ significantly under varying conditions. Further subgroup analysis was also conducted based on assessment tools, primary endpoints, and patient groups, and detailed results were provided in Data S2.

Issues related to the quality of RCTs in this field also require further consideration. While most studies implemented random allocation, many did not describe allocation concealment. Additionally, although assessors were generally blinded, patients might have directly perceived interventions like PES or taste, potentially introducing bias. Furthermore, most studies were registered before trial initiation, but seven out of 19 articles did not report registration information.

### 4.3 Effects and Potential Mechanisms of Oropharyngeal Sensory Stimulations

Sensory stimulation can improve swallowing function by inducing changes at both central and peripheral levels. Sensory input to the swallowing central nervous system arises from stimulation of the oral, pharyngeal, and laryngeal mucosa, which respond to stimuli such as touch, temperature, taste, and vibration [10]. At the central level, sensory stimulation drives long‐term changes in motor areas of the cerebral cortex, including increased excitability and representation area for the pharynx. Electroencephalography (EEG) studies have shown enhanced motor‐evoked and sensory‐evoked potentials after PES and capsaicin application [22]. Magnetoencephalographic evidence indicates improved swallowing processing efficiency, with attenuation of event‐related desynchronization in sensorimotor brain areas [50]. Functional near‐infrared spectroscopy (fNIRS) and functional magnetic resonance imaging (fMRI) studies have demonstrated a dramatic impact on both the sensory and motor cortex [51, 52]. At the peripheral level, interventions like PES and capsaicin increase salivary substance P levels, facilitating the swallow reflex [53, 54]. These mechanisms underline the potential for sensory stimulation to promote recovery in ND.

Thirteen clinical studies included in this meta‐analysis applied PES, while one used stimulation on the faucial pillar. Jayasekeran et al. identified the optimal stimulation protocol as 10 min per day for three consecutive days [28], which has since been widely adopted. In addition to the effects on swallowing function, the current meta‐analysis on PES effects in post‐stroke tracheostomy patients with severe dysphagia included two studies demonstrating significant benefits in accelerating decannulation. Tracheostomy is often necessitated by prolonged inability to breathe or protect the airway, with severe dysphagia increasing risks of aspiration and reflux [55].

For capsaicin, although no significant effect was noticed with the subgroup analysis, it remains a promising treatment for ND. Capsaicin has been shown in previous studies to improve swallowing efficacy by increasing spontaneous swallowing frequency [56], pharyngeal contractile strength, and upper esophageal sphincter function [53]. Additionally, capsaicin enhances swallowing safety by stimulating the swallowing reflex, strengthening cough, and reducing post‐swallow residue in stroke patients [57].

Beyond PES and capsaicin, other sensory stimulation approaches have shown potential but were not included in the current meta‐analysis due to the inclusion criteria, lack of data, and the study design. Taste stimulation using citric acid or strong‐flavored boluses has been shown to improve swallowing physiology by altering the timing or amplitude of swallowing movements [58], thereby increasing safety and efficiency [59]. Air pressure or mechanical stimulation of glossopharyngeal nerve afferents significantly increases swallowing frequency [60]. Similarly, carbonated fluids strongly stimulate reflexogenic areas of the larynx, reducing aspiration and improving upper airway protection [61]. These methods highlight the diverse ways sensory stimulation can enhance swallowing recovery. However, high‐quality RCTs for those strategies remain limited.

In addition, neurogenic diseases, such as stroke, are often accompanied by cognitive dysfunction, which can affect sensory processing and compliance with sensory stimulation, thereby impacting swallowing rehabilitation [62]. Future research should also consider integrating cognitive function assessment in swallowing studies and, when necessary, explore whether sensory stimulation combined with cognitive training [63] may produce better outcomes.

### 4.4 Limitations of the Review and the Future

The current review has several limitations. First, most studies recruited patients after stroke, which might limit generalizability to other ND populations. Most studies included in the meta‐analysis applied electrical stimulation, which may limit generalizability to other sensory stimulations. Second, the overall quality of evidence was generally medium based on the risk of bias analysis, predominantly because of the difficulty in blinding sensory interventions. The results were also limited by substantial heterogeneity. Finally, some studies did not adequately report outcome data. However, we extracted necessary data from figures or requested it from authors. Further trials with larger sample sizes across varied interventions are needed.

## 5 Conclusion

In conclusion, this systematic review and meta‐analysis highlights the therapeutic potential of oropharyngeal sensory stimulation for ND. The findings demonstrate significant benefits for swallowing function and decannulation, particularly with PES, while capsaicin and other sensory stimulations remain promising but clinical evidence supported with RCTs remains sparse. Despite these positive outcomes, the results should be interpreted with caution considering the heterogeneity among studies, moderate quality of evidence, and small sample size. Future research with larger sample sizes is warranted to further investigate the clinical application. Particular attention should be given to the exploration of capsaicin and other gustatory stimulation methods in treatment protocols, given the current limitations in the evidence.

## Author Contributions

**Meng Dai:** conceptualization (equal), data curation (equal), formal analysis (lead), roles/writing – original draft (lead), and writing – review and editing (equal). **Ivy Cheng:** data curation (equal) and writing – review and editing (equal). **Ayodele Sasegbon:** data curation (equal), and writing – review and editing (equal). **Wanqi Li:** data curation (equal) and writing – review and editing (equal). **Shaheen Hamdy:** conceptualization (equal), writing – review and editing (equal), and supervision (lead).

## Conflicts of Interest

Shaheen Hamdy is the chief scientific officer and stocks/shares holder of Phagenesis Ltd., a company involved in neuromodulatory dysphagia treatment. Other authors declare that there is no conflicts of interest.

## Supporting information


Data S1.

Data S2.

## Data Availability

This study is a meta‐analysis that utilized data extracted from previously published studies. All data used in this analysis are available in the original publications. No new primary data were generated or collected for this study.
