# Supplementary material for: A Meta‐Analysis and Systematic Review of the Effects of Sensory Modulation Treatments for Neurogenic Oropharyngeal Dysphagia
Source: CNS Neurosci Ther. 2025 Jul 8;31(7):e70452. doi: 10.1111/cns.70452 (PMC12235329; doi:10.1111/cns.70452)
Supplement: Supplementary file 1 — Data S1. [file CNS-31-e70452-s001.docx]

**Sensory stimulation targeting neurogenic dysphagia:**

**A systematic review**

P: Neurogenic dysphagia

I: sensory related interventions

C: TBD whether whether the non-controlled study should be included

O: instant outcomes from clinical assessment/VFSS/FEES; safety/efficacy of swallowing functions in clinical trials

**SEARCH TERMS**

**PUBMED 475**

TITLE AND ABSTRACT = (Deglutition OR Deglutition Disorders OR swallow* OR dysphag*)

AND

TITLE AND ABSTRACT = (Sensory OR Thermal OR Tactile OR Chemical OR Electrical OR Mechanical OR Taste OR Gustatory OR Carbona* OR Air pulse OR Capsaici* OR TRP OR TRPV OR TRPA OR TRPM OR vanilloid receptor agonist OR Sweet OR Sour OR taste OR bitter OR salt* OR temperature OR cold OR hot OR lemon OR pepper OR citr* OR acid OR acetic OR isopulegol OR menthol OR airpuffs OR flavor OR Piperin*)

Filters: Full text,( Clinical Trial, Controlled Clinical Trial, Randomized Controlled Trial), Humans, English, Adult: 19+ years

**Cochrane 969**

Title Abstract Keywords = (Deglutition OR Deglutition Disorders OR swallow* OR dysphag*)

AND

Title Abstract Keywords = (Sensory OR Thermal OR Tactile OR Chemical OR Electrical OR Mechanical OR Taste OR Gustatory OR Carbona* OR Air pulse OR Capsaici* OR TRP OR TRPV OR TRPA OR TRPM OR vanilloid receptor agonist OR Sweet OR Sour OR taste OR bitter OR salt* OR temperature OR cold OR hot OR lemon OR pepper OR citr* OR acid OR acetic OR isopulegol OR menthol OR airpuffs OR flavor OR Piperin*)

AND

Title Abstract Keywords = (patients* and adult)

Filters: English

**Embase via Ovid 1470**

(Deglutition OR Deglutition Disorders OR swallow* OR dysphag*).ab,ti.

AND

(Sensory OR Thermal OR Tactile OR Chemical OR Electrical OR Mechanical OR Taste OR Gustatory OR Carbona* OR Air pulse OR Capsaici* OR TRP OR TRPV OR TRPA OR TRPM OR vanilloid receptor agonist OR Sweet OR Sour OR taste OR bitter OR salt* OR temperature OR cold OR hot OR lemon OR pepper OR citr* OR acid OR acetic OR isopulegol OR menthol OR airpuffs OR flavor OR Piperin*).ab,ti.

Filter: English language； full text； humans； (article or article in press)

**Cinahl 203**

SU = (Deglutition OR Deglutition Disorders OR swallow* OR dysphag*) AND (Sensory OR Thermal OR Tactile OR Chemical OR Electrical OR Mechanical OR Taste OR Gustatory OR Carbona* OR Air pulse OR Capsaici* OR TRP OR TRPV OR TRPA OR TRPM OR vanilloid receptor agonist OR Sweet OR Sour OR taste OR bitter OR salt* OR temperature OR cold OR hot OR lemon OR pepper OR citr* OR acid OR acetic OR isopulegol OR menthol OR airpuffs OR flavor OR Piperin*)

Filter: English; All adult

**WOS 443**

(TI= (Deglutition OR Deglutition Disorders OR swallow* OR dysphag*) AND (Sensory OR Thermal OR Tactile OR Chemical OR Electrical OR Mechanical OR Taste OR Gustatory OR Carbona* OR Air pulse OR Capsaici* OR TRP OR TRPV OR TRPA OR TRPM OR vanilloid receptor agonist OR Sweet OR Sour OR taste OR bitter OR salt* OR temperature OR cold OR hot OR lemon OR pepper OR citr* OR acid OR acetic OR isopulegol OR menthol OR airpuffs OR flavor OR Piperin*) and (patient* and adult)))

OR

AB= (Deglutition OR Deglutition Disorders OR swallow* OR dysphag*) AND (Sensory OR Thermal OR Tactile OR Chemical OR Electrical OR Mechanical OR Taste OR Gustatory OR Carbona* OR Air pulse OR Capsaici* OR TRP OR TRPV OR TRPA OR TRPM OR vanilloid receptor agonist OR Sweet OR Sour OR taste OR bitter OR salt* OR temperature OR cold OR hot OR lemon OR pepper OR citr* OR acid OR acetic OR isopulegol OR menthol OR airpuffs OR flavor OR Piperin*))

**AND**

**(patient* and adult)**

Filter: English (Languages) and Article (Document Types)
