# Supplementary material for: A Meta‐Analysis and Systematic Review of the Effects of Sensory Modulation Treatments for Neurogenic Oropharyngeal Dysphagia
Source: CNS Neurosci Ther. 2025 Jul 8;31(7):e70452. doi: 10.1111/cns.70452 (PMC12235329; doi:10.1111/cns.70452)
Supplement: Supplementary file 2 — Data S2. [file CNS-31-e70452-s002.docx]

Sensitivity test - Subgroup analysis

To further explore the origin of the heterogeneity, we did subgroup analyses in Review Manager online (<https://revman.cochrane.org/info>) and the results are as follows. As shown in Figure S1, subgroup analysis based on the primary endpoints indicated non-significant immediate effects (n = 5, SMD [95% CI] = 0.19 [-0.37, 0.75], *p* = 0.40; *I²* = 6%), borderline significant improvement within one week (n = 4, SMD [95% CI] = 1.03 [0.05, 2.01], *p* = 0.05; *I²* = 6%), and sustained benefits at 2-3 weeks (n = 8, SMD [95% CI] = 1.01 [0.32, 1.70], *p* = 0.01; I² = 83%). For different patient groups (Figure S2), subgroup analysis revealed improvements in stroke patients: stroke patients without tracheostomy (n = 11, SMD [95% CI] = 0.63 [0.16, 1.11], *p* = 0.01; *I²* = 73%), stroke patients with tracheostomy (n = 3, SMD [95% CI] = 1.03 [0.05, 2.01], *p* = 0.05; *I²* = 6%). However, no significant benefits were found among patients with other neurodegenerative conditions (n = 3, SMD [95% CI] = 1.39 [-2.04, 4.82], *p* = 0.22; *I²* = 77%). Analysis by assessment method (Figure S3) demonstrated significant variation in measured outcomes. PAS-based assessments showed borderline significant moderate effects (n = 10, SMD [95% CI] = 0.70 [0.00, 1.41], *p* = 0.05; *I²* = 76%), while results were not statistically significant for DSRS (n = 2, SMD [95% CI] = 0.44 [-4.12, 5.00], *p* = 0.44; *I²* = 47%) and SSA (n = 2, SMD [95% CI] = 1.27 [-0.28, 2.81], *p* = 0.06; *I²* = 0%). FEES-based assessments demonstrated stronger effects (n = 3, SMD [95% CI] = 1.03 [0.05, 2.01], *p* = 0.05; *I²* = 6%).


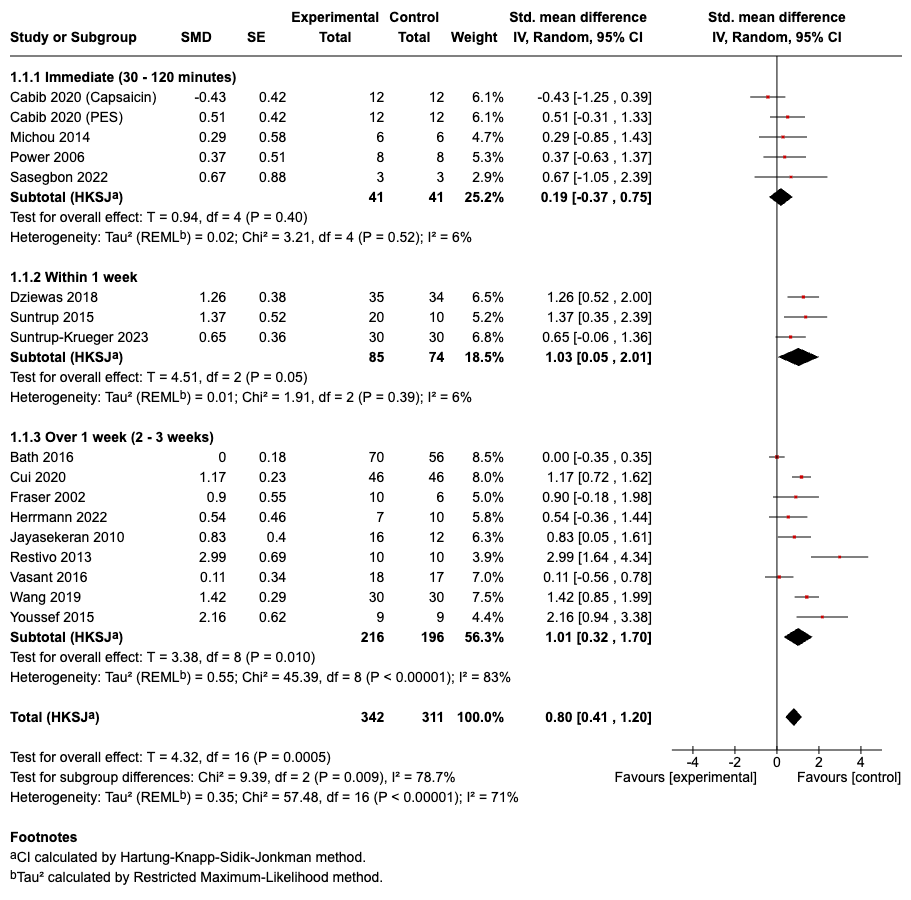


Figure S1. Subgroup analysis based on *different primary endpoints after intervention.*


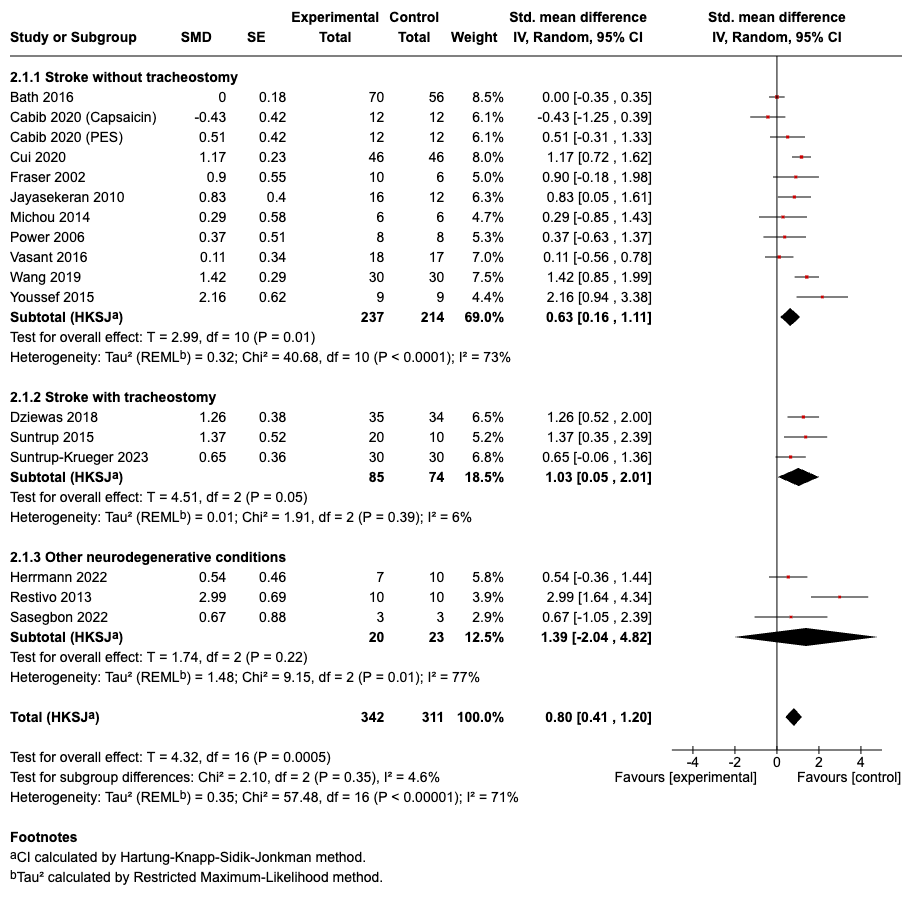


Figure S2. Subgroup analysis based on *different patient groups.*


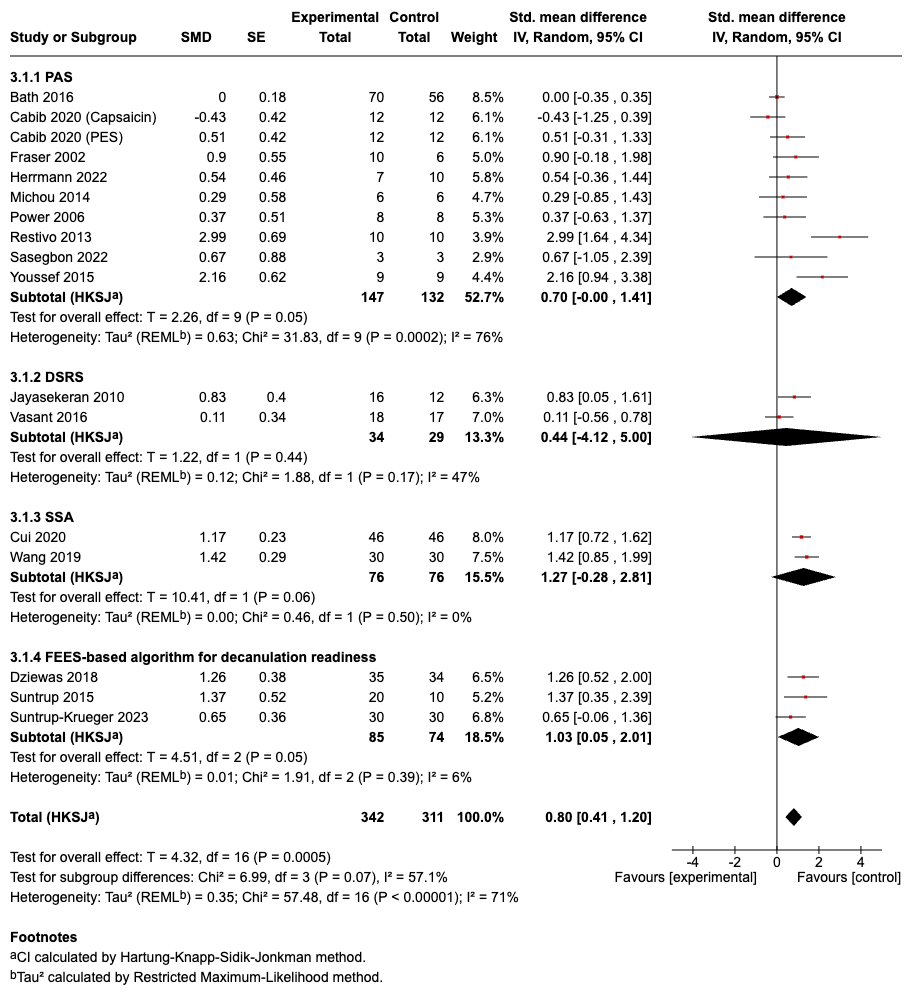


Figure S3. Subgroup analysis based on *different swallowing assessment methods.* PAS - penetration and aspiration scale, DSRS - Dysphagia Severity Rating Scale, SSA - Swallowing Safety Assessment, FEES - fiberoptic endoscopic evaluation of swallowing.
